# Gastric metastasis from hormone receptor–positive breast cancer ten years after radical mastectomy: a case report and literature review

**DOI:** 10.3389/fonc.2026.1750684

**Published:** 2026-05-15

**Authors:** Qiang Zhan, Gao Wang, Hongxia Wan

**Affiliations:** 1 Department of Breast Surgery, Pingxiang People’s Hospital, Pingxiang, China; 2 Fuzhou Medical College of Nanchang University, Fuzhou, China

**Keywords:** breast neoplasms, cyclin-dependent kinase inhibitors, diagnostic errors, neoplasm metastasis, stomach neoplasms

## Abstract

Breast cancer is the most common malignancy among women, whereas gastrointestinal metastasis is uncommon and gastric involvement is particularly rare. We report a 59-year-old woman who presented with epigastric discomfort and dysphagia 10 years after surgery and postoperative chemotherapy for right breast cancer. Gastroscopy and histopathological examination with immunohistochemistry confirmed hormone receptor–positive, HER2-negative metastatic breast carcinoma involving the stomach, and staging evaluation also revealed multiple bone metastases. Because the clinical manifestations closely mimicked primary gastric cancer, the diagnosis was challenging and required careful clinicopathological correlation. This case highlights the importance of considering late gastrointestinal metastasis in long-term breast cancer survivors and underscores the critical role of endoscopic biopsy, immunohistochemistry, and multidisciplinary evaluation in establishing an accurate diagnosis. Treatment with a CDK4/6 inhibitor combined with an aromatase inhibitor resulted in rapid symptom relief and short-term disease stabilization.

## Introduction

Breast cancer remains the most common malignancy and the leading cause of cancer-related mortality among women worldwide (1, 2). The most frequent metastatic sites include the bone, lung, liver, and brain, whereas gastrointestinal metastases are uncommon (3, 4). Among gastrointestinal sites, gastric involvement is particularly rare, accounting for approximately 0.3–2% of metastatic breast cancer cases (5). Although uncommon, gastric metastasis is clinically important because it may present with nonspecific gastrointestinal symptoms and can be mistaken for a primary gastric malignancy.

The pattern of gastrointestinal spread differs according to histologic subtype. Most reported cases of gastric metastasis arise from invasive lobular carcinoma (ILC), likely because loss of E-cadherin facilitates diffuse infiltration of the gastric wall (4, 6, 7). By contrast, invasive ductal carcinoma (IDC), despite being the most common histologic subtype of breast cancer, is much less frequently reported to metastasize to the stomach (7). This distinction is diagnostically relevant, because gastric metastasis from IDC may be unexpected in routine clinical practice and therefore more likely to be misinterpreted as primary gastric adenocarcinoma.

In addition, hormone receptor–positive breast cancer is characterized by a prolonged natural history and a persistent risk of late recurrence, particularly in some patients after a long disease-free interval (8).

## Case presentation

### Initial breast cancer diagnosis and treatment (10 years prior)

Ten years prior to the current presentation, the patient underwent right modified radical mastectomy for right breast cancer at a maternal and child health hospital in Jiangxi Province, followed by postoperative chemotherapy at the same hospital. However, because the complete original clinical and pathological records were not available to us, detailed information regarding the initial tumor stage, full pathological findings, immunohistochemical profile, and some treatment details could not be fully reviewed in the present report.

### Current presentation

A 59-year-old woman presented with a one-month history of upper abdominal discomfort and progressive dysphagia. She had undergone a right radical mastectomy for breast carcinoma ten years earlier, with no family history of breast or gastrointestinal malignancy. The patient reported mild epigastric pain, aggravated by eating, and early satiety without hematemesis or melena. Mild weight loss was initially noted. Physical examination revealed mild tenderness in the epigastric region without palpable masses, hepatosplenomegaly, or lymphadenopathy. Bowel sounds were normal (4–5 times/min).

### Patient perspective

The patient reported that her epigastric discomfort and dysphagia had progressively worsened over approximately four weeks, significantly interfering with her ability to eat regular meals and affecting her daily quality of life. She initially attributed these symptoms to a benign gastrointestinal condition but became increasingly concerned as they progressed. Upon receiving the diagnosis of metastatic breast cancer involving the stomach, 10 years after mastectomy, she experienced considerable emotional distress and disbelief. After multidisciplinary counselling and initiation of ribociclib plus letrozole, she reported a marked improvement in dysphagia and epigastric symptoms within two to three weeks, with better oral intake and meaningful symptomatic relief. However, no further patient-reported information was available after later disease progression, because telephone follow-up in April 2026 was declined.

### Diagnostic workup

**Figure 1:**
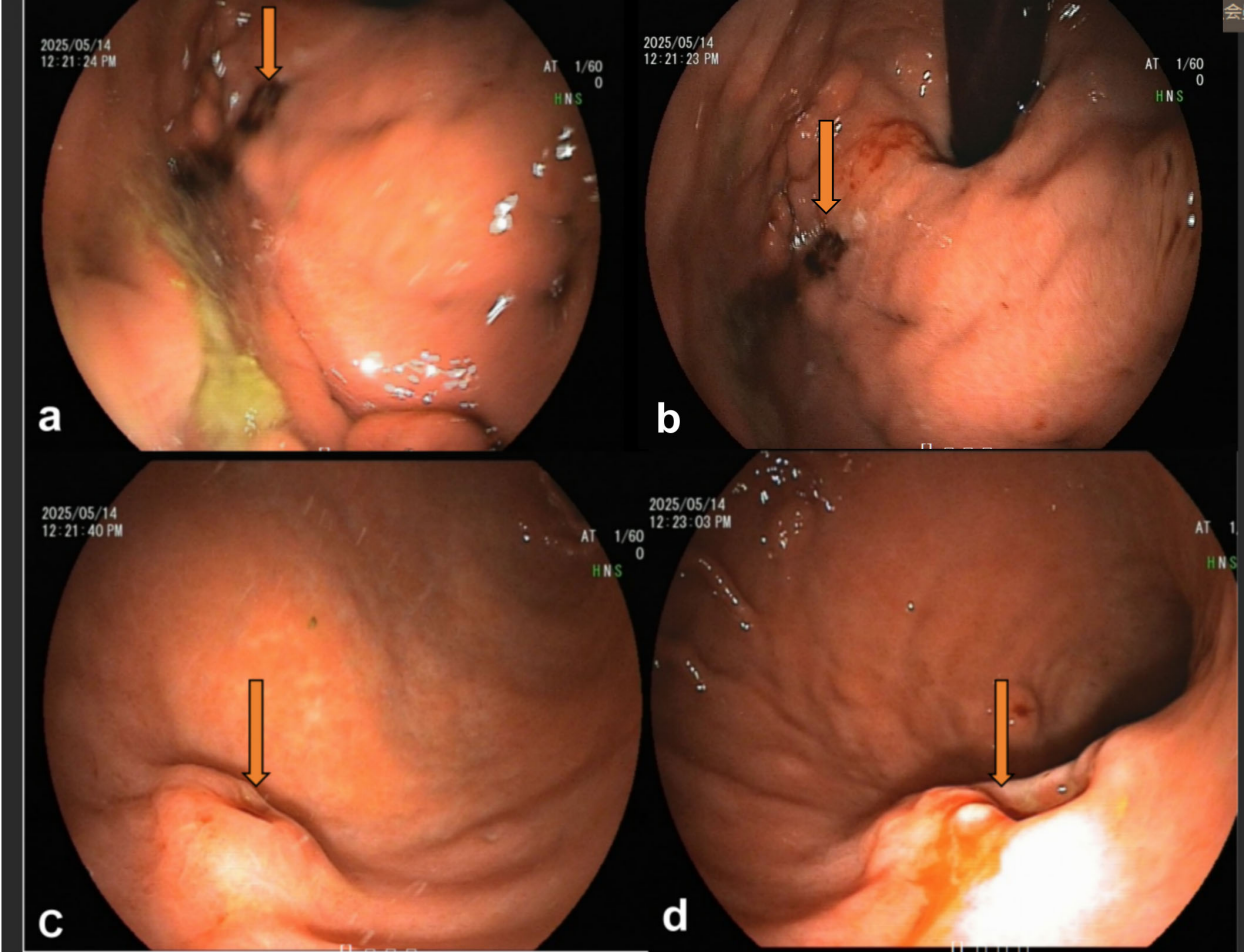
Gastroscopy revealed: **(a, b)** Ulcer at the gastric fundus near the cardia, with a dirty surface and elevated surrounding mucosa; **(c, d)** epressed ulcer at the mid-body of the stomach near the posterior wall, covered with black fur, and the surrounding mucosa showing crater-like elevation.

Contrast-enhanced chest CT demonstrated postoperative changes in the right breast without evidence of local recurrence. Gastroscopy revealed nodular lesions in the gastric fundus with mucosal erosion and easy bleeding upon biopsy (**Figures 1a, b**). Additional lesions were seen in the gastric body, characterized by edematous mucosa and an ulcerative lesion with a “volcano-like” elevation approximately 12 × 10 mm in size (**Figures 1c, d**).

**Figure 2:**
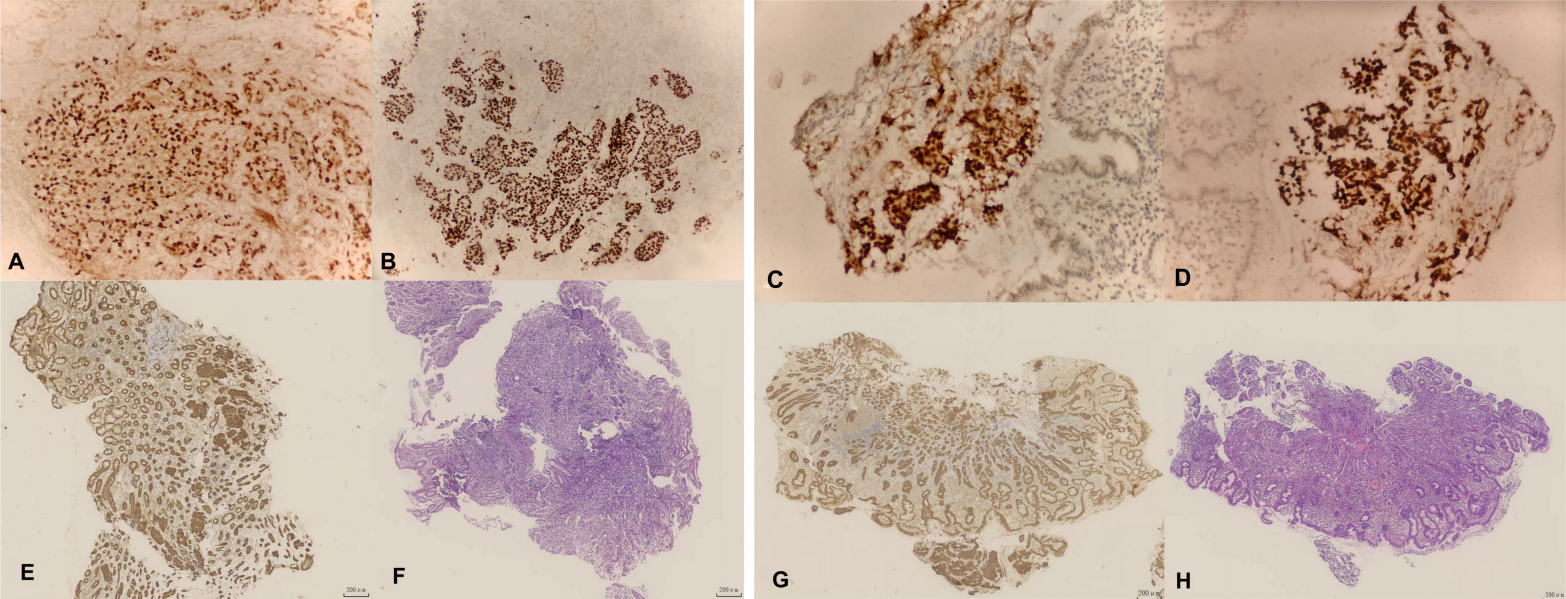
IHC examination: **(A)** ER expression in gastric fundus-2 **(B)** GATA-3 expression in gastric fundus-2 **(C)** ER expression in gastric body-1 **(D)** GATA-3 expression in gastric body-1 **(E)** CK expression in gastric fundus **(F)** Lesion in gastric fundus **(G)** CK expression in gastric body **(H)** Lesion in gastric body.

**Figure 3:**
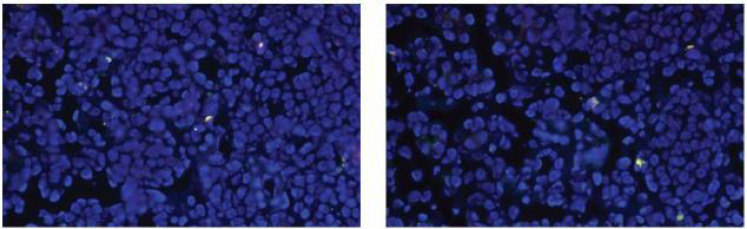
FISH expression in: tumor cells, the ratio of HER2 gene copy number to the centromere reference signal copy number of chromosome 17 was less than 2.0. No gene amplification was detected, and FISH negativity indicated no overexpression of HER2 protein.

Histopathology showed poorly differentiated adenocarcinoma consistent with metastatic disease. Immunohistochemistry demonstrated positivity for cytokeratin (CK), estrogen receptor (ER, ≈ 90%), and GATA3, with negative results for CD68, synaptophysin, and chromogranin A (**Figure 2**). Ki-67 proliferation index was approximately 2%. Progesterone receptor (PR) was negative, HER2 immunostaining was 2+, but FISH testing revealed no HER2 gene amplification. PD-L1 combined positive score (CPS)was 2 (**Figure 3**).

**Figure 4:**
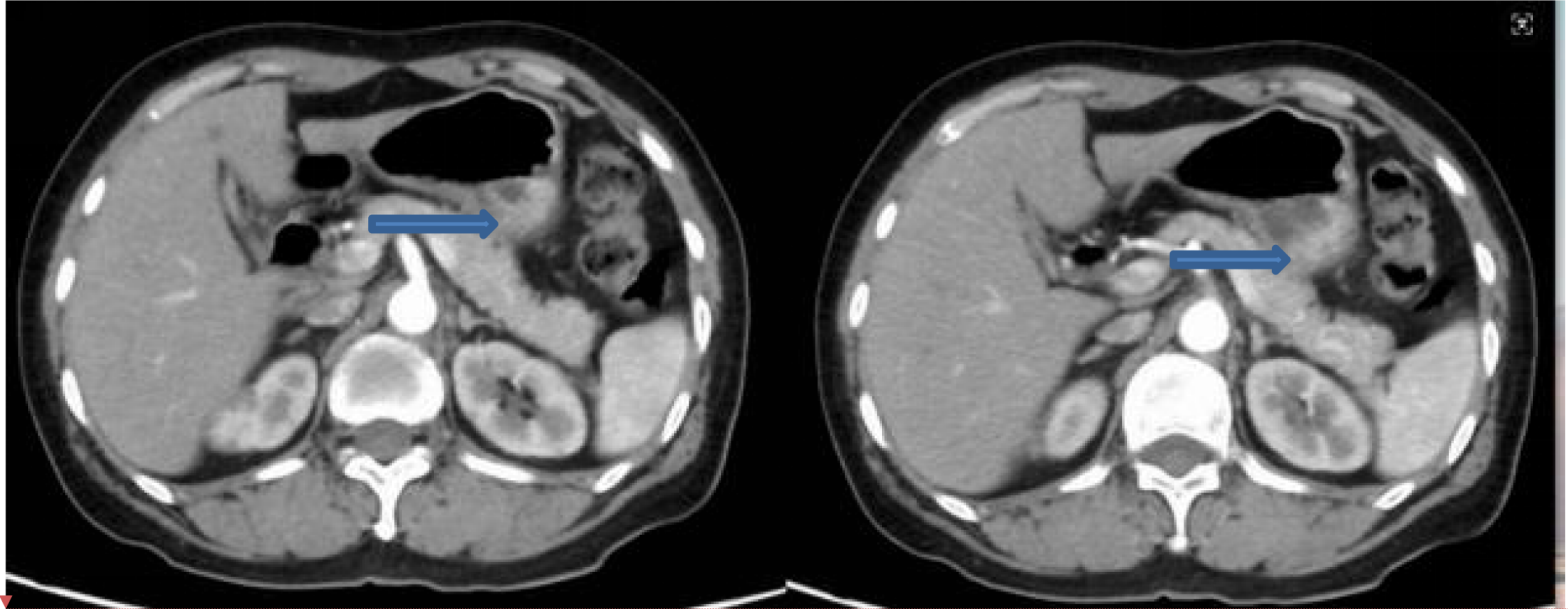
Representative axial contrast-enhanced abdominal CT images obtained at admission for recurrent disease, showing thickening of the gastric wall along the greater curvature with heterogeneous enhancement.

A whole-body bone scan showed multiple areas of increased radionuclide uptake, consistent with widespread osseous metastases. Cervical CT demonstrated multiple mildly enlarged lymph nodes in the bilateral cervical and mediastinal regions, as well as small nodules in both upper lung lobes. Brain MRI identified an abnormal signal focus in the left frontal region, which was considered more suggestive of a meningioma than metastatic disease. Contrast-enhanced abdominal CT obtained at admission for recurrent disease showed thickening of the gastric wall along the greater curvature with heterogeneous enhancement (**Figure 4**). Following multidisciplinary team (MDT) discussion, the final diagnosis was metastatic breast carcinoma with gastric and bone involvement as summarized in **Table 1**.

### Clinical timeline

**Table 1:** Clinical timeline of the patient’s initial breast cancer treatment, recurrent presentation, diagnosis, and management.

Time point	Event	Findings/interventions
10 years prior	Initial breast cancer treatment	Right breast cancer treated with right modified radical mastectomy at a maternal and child health hospital in Jiangxi Province
After surgery	Postoperative treatment	Postoperative FEC chemotherapy (4 cycles) was administered at the same hospital
1 month before admission	Symptom onset	Progressive dysphagia, epigastric discomfort, and early satiety
At admission	Gastroscopy	Nodular lesions in the gastric fundus and additional lesions in the gastric body with ulcerative changes
At admission	Pathology and immunohistochemistry	Poorly differentiated adenocarcinoma; ER-positive, GATA3-positive, PR-negative; HER2 IHC 2+ with no HER2 amplification on FISH; Ki-67 approximately 2%
At admission	Staging evaluation	Multiple bone metastases; small nodules in both upper lung lobes; abnormal left frontal lesion considered more suggestive of meningioma than metastasis
After MDT discussion	Final diagnosis	Metastatic breast carcinoma with gastric and bone involvement
Day 1 of treatment	Systemic treatment initiation	Ribociclib plus letrozole; ibandronate sodium for bone protection
4 weeks after treatment initiation	Early response	Marked improvement in dysphagia and epigastric discomfort; improved oral intake
May 29, 2025	Before discharge	Symptomatic improvement
November 22, 2025	Latest documented clinical reassessment	Disease progression was documented
April 2026	Telephone follow-up attempt	Further follow-up was attempted, but the patient declined interview; no additional post-progression information was available

### Treatment

Given the ER-positive, HER2-non-amplified phenotype of the metastatic lesion, the patient received combination endocrine therapy with the CDK4/6 inhibitor ribociclib and the aromatase inhibitor letrozole, along with ibandronate sodium for bone protection. Supportive therapy included vitamin B complex and mecobalamin for symptoms related to right recurrent laryngeal nerve palsy. After four weeks of treatment, the patient’s dysphagia and epigastric discomfort markedly improved, and she tolerated oral intake well. On May 29, 2025, before discharge, the patient reported that her symptoms had improved following treatment. However, disease progression was documented at the latest available clinical reassessment on November 22, 2025. Further telephone follow-up was attempted in April 2026, but the patient declined interview; therefore, no additional post-progression information was available.

## Discussion

Gastric metastasis from breast carcinoma is rare and poses a substantial diagnostic challenge. Although breast cancer most commonly metastasizes to the bone, lung, liver, and brain, gastrointestinal involvement is uncommon, and gastric localization has been reported in only a small proportion of metastatic cases (4, 9). In published series, gastric metastases are more frequently associated with invasive lobular carcinoma than with invasive ductal carcinoma, likely because of the characteristic infiltrative growth pattern of lobular tumors (7). Regardless of histologic subtype, however, gastric metastasis is clinically important because its manifestations are often nonspecific and may closely resemble those of primary gastric malignancy (10–13).

The present case is clinically instructive for several reasons. First, metastatic recurrence occurred 10 years after surgery for breast cancer, highlighting the possibility of very late relapse in breast cancer survivors. Second, the patient presented with epigastric discomfort, dysphagia, and endoscopic gastric lesions that closely mimicked primary gastric cancer. Third, the diagnosis was established only after clinicopathological correlation and immunohistochemical evaluation, emphasizing the importance of considering metastatic breast carcinoma in the differential diagnosis of gastric lesions in patients with a prior history of breast cancer. These features underscore the educational value of the case despite the absence of therapeutic novelty (10–14). Histopathological and immunohistochemical evaluation remains central to differentiating metastatic breast carcinoma from primary gastric adenocarcinoma. Morphologic assessment alone may be insufficient, particularly when gastric lesions are poorly differentiated or infiltrative (4, 13). In general, a breast origin is supported by expression of markers such as ER, CK7, and GATA3, together with the absence of gastrointestinal markers such as CK20 and CDX2 (15). In the present case, strong ER and GATA3 expression, together with the absence of HER2 amplification on FISH, supported a breast origin. Because false-negative biopsy results may occur owing to submucosal spread or patchy tumor distribution, repeated and sufficiently deep biopsies are important when the initial findings are inconclusive (4).

From a therapeutic perspective, systemic therapy remains the mainstay of treatment for gastric metastasis from breast cancer, whereas surgery is generally reserved for selected palliative indications such as bleeding, obstruction, or perforation (16). For metastatic breast carcinoma with an ER-positive, HER2-non-amplified phenotype, endocrine therapy combined with a CDK4/6 inhibitor is an established treatment strategy (17, 18). In our patient, ribociclib plus letrozole was associated with rapid symptomatic improvement, and ibandronate was administered to preserve bone health.

However, disease progression was documented at the latest available clinical reassessment on November 22, 2025. Further telephone follow-up in April 2026 was declined, and therefore no additional post-progression clinical information was available.

This case highlights several practical clinical messages. Clinicians should maintain suspicion for metastatic breast carcinoma when long-term breast cancer survivors develop new gastrointestinal symptoms, even after a prolonged disease-free interval. Accurate diagnosis requires close integration of prior oncologic history, endoscopic findings, pathology, and immunohistochemistry. Multidisciplinary evaluation is essential to avoid misdiagnosis and to guide appropriate systemic treatment in this uncommon but clinically significant pattern of recurrence.

## Conclusion

This case highlights three main clinical lessons. First, clinicians should maintain awareness of the possibility of late metastatic recurrence in long-term breast cancer survivors, particularly when new gastrointestinal symptoms arise many years after initial treatment. Second, accurate diagnosis of gastric metastasis from breast carcinoma requires close clinicopathological correlation and appropriate immunohistochemical evaluation, particularly with markers such as ER and GATA3, to distinguish metastatic disease from primary gastric malignancy. Third, once metastatic breast carcinoma is confirmed, multidisciplinary evaluation is essential to guide individualized systemic treatment. In the present case, endocrine-based therapy combined with a CDK4/6 inhibitor was associated with early symptomatic improvement and short-term disease stabilization.

## Limitations

This case report has several limitations. First, long-term outcome data remain limited. Although the patient experienced early symptomatic improvement and short-term disease stabilization, disease progression was documented on November 22, 2025, and further telephone follow-up in April 2026 was declined. Second, because the patient’s initial treatment was performed at a maternal and child health hospital in Jiangxi Province, the complete original clinicopathological records were not available for full review. Therefore, formal staging, detailed pathological findings, and the original immunohistochemical profile of the primary tumor could not be fully verified.

## Data Availability

The original contributions presented in the study are included in the article/supplementary material. Further inquiries can be directed to the corresponding author.
